# LXGB: a machine learning algorithm for estimating the discharge coefficient of pseudo-cosine labyrinth weir

**DOI:** 10.1038/s41598-023-39272-6

**Published:** 2023-07-29

**Authors:** Somayeh Emami, Hojjat Emami, Javad Parsa

**Affiliations:** 1 Department of Water Engineering, University of Tabriz, Tabriz, 5971982284 Iran; 2 Department of Computer Engineering, University of Bonab, Bonab, Iran; 3 Department of Water Engineering, University of Tabriz, Tabriz, Iran

**Keywords:** Engineering, Hydrology

## Abstract

One of the practical and financial solutions to increase the efficiency of weirs is to modify the geometry of the plan and increase the length of the weir to a specific width. This increases the discharge coefficient (*C*_*d*_) of the weir. In this study, a new weir referred to pseudo-cosine labyrinth weir (PCLW) was introduced. A hybrid machine learning LXGB algorithm was introduced to estimate the *C*_*d*_ of the PCLW. The LXGB is a combination of the linear population size reduction history-based adaptive differential evolution (LSHADE) and extreme gradient boosting (XGB) algorithm. Seven different input scenarios were presented to estimate the discharge coefficient of the PCLW weir. To train and test the proposed method, 132 data series, including geometric and hydraulic parameters from PCLW1 and PCLW2 models were used. The root mean square error (*RMSE*), relative root mean square error (*RRMSE*), and Nash–Sutcliffe model efficiency coefficient (*NSE*) indices were used to evaluate the proposed approach. The results showed that the input variables were the ratio of the radius to the weir height (*R/W*), the ratio of the length of the weir to the weir height (*L/W*), and the ratio of the hydraulic head to the weir height (*H/W*), with the average values of *RMSE* = 0.009, *RRMSE* = 0.010, and *NSE* = 0.977 provided better results in estimating the *C*_*d* _of PCLW1 and PCLW2 models. The improvement compared to SAELM, ANFIS-FFA, GEP, and ANN in terms of *R*^2^ is 2.06%, 3.09%, 1.03%, and 5.15%. In general, intelligent hybrid approaches can be introduced as the most suitable method for estimating the *C*_*d*_ of PCLW weirs.

## Introduction

One of the main concerns of hydraulic engineers is the optimal management of limited water resources, in Iran. The ever-increasing growth of national investment in water projects leads to the optimization of water control and management projects in order to save national capital^1–3^. In recent years, hydraulic engineers have tried to measure the discharge with proper accuracy by building and installing measuring structures in the channels. One of the common structures in many dams and water transfer channels are labyrinth weirs, which are used for draining, measuring, and controlling the water level^4, 5^. These types of weirs are among the most practical surface structures, which have recently attracted the attention of various researchers. The pseudo-cosine labyrinth weirs (PCLW) with a long crown have a suitable performance for regulating the water level compared to other weirs. Numerous parameters are effective in determining the *C*_*d*_ in labyrinth weir with different plans. These parameters are related to several factors, including the upstream total hydraulic head (*H*_*u*_), downstream hydraulic head (*H*_*d*_), weir height (*W*), radius (*R*), number of cycles (*N*), shape of the weir crest (*CR*), collision of nape (*Na*), the approach flow conditions (*AF*), etc.^4^. Nowadays, several issues, including the increase in costs, time-consuming, and the occurrence of human error, have led to the use of 3D and computer models^6, 7^. Since manual calculations may involve human error, it is necessary to use novel intelligent methods such as meta-heuristic algorithms, artificial neural networks, fuzzy logic, etc. Several studies have been carried out by researchers in the investigation of the *C*_*d*_ of labyrinth weirs^8–15^. Considering some structural limitations (such as structure dimensions and weir angle) and using classical calculation methods such as linear and non-linear regression methods, the researchers have determined the *C*_*d*_ of weirs.

Azamathulla and Wu^16^ used the support vector machine (SVM) to accurately estimate the longitudinal dispersion coefficients in natural rivers. With a test on real-world datasets, the SVM algorithm is proven to generate encouraging results. In another work, Azamathulla et al.^17^ proposed SVM to estimate the *C*_*d*_ in side weirs. The experimental results proved the superiority of the SVM compared with counterpart adaptive neuro-fuzzy inference systems (ANFIS) and artificial neural networks (ANNs). Bilhan et al.^18^ estimate the *C*_*d* _of labyrinth weirs using support vector regression (SVR) and an outlier robust extreme learning machine. The results showed that machine learning methods estimated the *C*_*d*_ values more accurately. Safarrazavizadeh et al.^19^ performed a laboratory investigation of the flow on labyrinth weirs with a semicircular and sinusoidal plan. Observations showed that the discharge coefficient in labyrinth weirs with a semicircular and sinusoidal plan, unlike linear weirs, has an upward trend in low water loads (*H*_*T*_*/P* < 0.35) and decreases after reaching its maximum value. Bonakdari et al.^20^ investigated the effectiveness of the gene expression programming (GEP) method for estimating *C*_*d*_. Results show that the GEP method provides better results in predicting *C*_*d*_. Shafiei et al.^21^ used the ANFIS-firefly algorithm (ANFIS-FFA) method to estimate the *C*_*d*_ of triangular labyrinth weirs. Results showed that the ANFIS-FFA model is more accurate in predicting the *C*_*d*_ of triangular labyrinth weirs. Emami et al.^8^ estimated the *C*_*d* _of W-planform labyrinth weirs using the improved self-adaptive differential evolutionary algorithm and support vector regression (ISaDE-SVR) method. ISaDE-SVR is highly effective in estimating the *C*_*d*_ of W-planform weirs. Norouzi et al.^22^ simulated *C*_*d*_ using a self-adaptive robust learning machine (SAELM) model. The results showed that the SAELM model estimated the *C*_*d*_ with high accuracy. Wang et al.^23^, investigated the application of genetic algorithm (GA), particle swarm optimization (PSO), and traditional BP neural network in predicting the *C*_*d*_ of triangular labyrinth weir. The results showed that GA-BPNN and PSO-BPNN methods have high efficiency in predicting *C*_*d*_. Chen et al.^24^ used SVM, random forest (RF), linear regression, SVM, k-nearest neighbor (KNN), and decision tree (DT) in predicting the *C*_*d*_ of streamlined weirs. Ahmad et al.^25^ used the ANN model to predict the *C*_*d*_ of an arced labyrinth side weir. The results indicated that *C*_*d*_ calculated by ANN is more accurate. Emami et al.^26^ used the Walnut algorithm and SVR method to predict the *C*_*d*_ of triangular labyrinth weirs. Safari et al.^27^ evaluated ANN, GEP, and regression models to estimate the *C*_*d*_ of the broad-crested weir. The results showed that ANN estimates the *C*_*d*_ better than GEP models and regression models.

In the previous studies, according to the many geometrical models that have been investigated by different researchers, the *C*_*d*_ of PCLW has not been investigated. Therefore, in the present study, by using the intelligent model of the differential evolution (LSHADE) and extreme gradient boosting (XGB) approach, the *C*_*d*_ of the PCLW was estimated. The proposed approach was investigated with different combinations of features to identify the high-performance combination of features.

The contributions of this paper are as follows:

- Introducing the LXGB algorithm, which integrates the LSHADE with XGB to tune the XGB parameters and further enhance its estimation performance.
- Using the LXGB algorithm to estimate the *C*_*d*_ of PCLW. The proposed algorithm models the
- Evaluating the proposed model with a real-world dataset and compared with state-of-the art algorithms. The experimental results show the superiority of the proposed method compared with counterparts in terms of performance measures.

The remaining sections of this study are organized as follows. Section "Material and methods" illustrates the experimental materials and the presented hybrid approach. Section "Results and Discussion" presents the results and discussions. Section "Conclusion" summarizes the paper and supplies recommendations for coming work.

## Material and methods

### Dimensional analysis

The 1-dimensional equation of the flow on the PCLW is as follows^28^:

1 $$Q = \frac{2}{3}\,\mathop C\nolimits_{d} \,L\,\sqrt {2g} \,\,\mathop {\mathop H\nolimits_{T} }\nolimits^{1.5}$$

where *Q* is the discharge, *g* shows the acceleration of gravity, *L* is the length of the weir, and *H*_*T*_ is the hydraulic height (*h* + *V*_*2*_*/*2* g*). The *C*_*d*_ of labyrinth weirs in free flow conditions depends on geometric and hydraulic parameters as follows:

2 $$C_{d} = \,f_{1} (B,\,L,\,H_{T} ,\,H_{d} ,\,V,W,\,R,\,S,\,t,\,\alpha ,\,N,\,g,\,\rho ,\,\mu ,\,\sigma ,\,CS,\,JS,\,SW)$$

where *B* is the channel width, *H*_*d*_ is the total hydraulic height (downstream of the weir), *V* shows the flow velocity, *W* indicates the height of the weir, *R* is the radius of weir curvature, *S* is the length of the straight part between the curves of the weir, *t* is the thickness of the weir, *α* represents the angle of the straight section between the weir curves with the direction of the channel, *N* indicates the number of cycles, *ρ* indicates the fluid density, *μ* the dynamic viscosity, *σ* shows the surface tension, *CS* means the shape of the weir crest, *JS* denotes the shape of the flowing blade, and *SW* represents the approaching flow and the sidewall effect.

Equation (2) can be written as follows:

3 $$C_{d} = \,f_{2} \,({\text{Re}} ,\,We,\,Fr,\,\frac{{H_{T} }}{W},\,\frac{{H_{d} }}{W},\,\frac{L}{W},\,\frac{B}{W},\,\frac{R}{W},\,\frac{S}{W},\frac{t}{W},\,\alpha ,\,N,\,CS,\,JS,\,SW)$$

where *Re* is the Reynolds number, *We* mean the Weber number, and *Fr* is the Froude number. Henderson^29^ concluded that if *Re* < 2000, the effect of viscosity can be neglected. Novak et al.^30^ concluded that if the water height on the weir is more than 3 to 4 cm, the effect of surface tension is ignored. Due to the turbulent flow and minimum water height of 5 cm on the weir, the impacts of the *Re* and *We* numbers were removed. The shape of the edge of all used weirs was selected as a sharp-crested, and the effect of *CS* was ignored. Due to the installation of weirs perpendicular to the main flow and the absence of local contraction at their installation location, the conditions of the approaching *SW* flow were considered the same for all experiments.

Equation (3). is simplified as the following equation:

4 $$C_{d} = \,f_{3} \,(\frac{{H_{T} }}{W},\,\frac{L}{W},\,\frac{B}{W},\,\frac{R}{W},\,\frac{S}{W},\,\frac{t}{W},\,\alpha ,\,N)$$

### Experimental models

**Figure 1:**
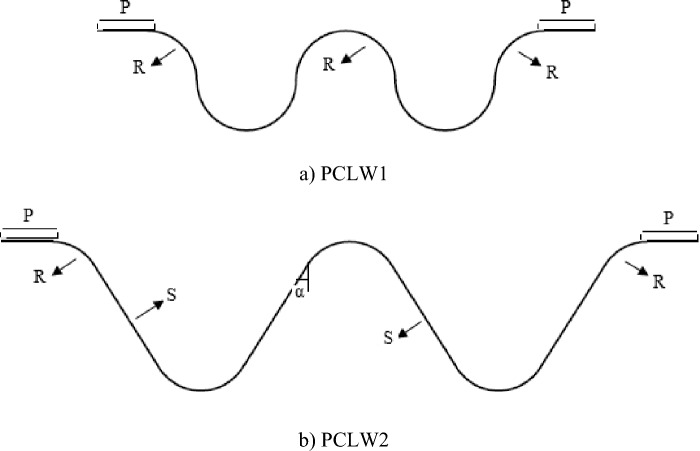
A big picture of PCLW1 and PLCW2.

The simulation of the flow around the PCLW was carried out in a channel with a width, length, and height of 0.49 m to 1.115 m, 3.2 m, and 0.5 m, respectively. In Fig. 1, the PCLW models and their geometric features are shown.

The geometric features and the range of experimental parameters of the PLCW are presented in Table 1.

**Table 1:** The geometric features of PLCW used in simulations.

Q (l/s)	H/W	α(°)	L(cm)	P(cm)	N	W(cm)	S(cm)	D(cm)
60–100	0.2–0.9	10	90–225	15.75	2–3	10–20	5–40	2.5–10

### Extreme gradient boosting (XGB)

XGB^31–33^ is a robust supervised learning solution to regression, classification, and ranking problems in a fast and accurate way. XGB is a more generalized form of gradient-boosting decision trees. It utilizes parallel processing, resolves missing values efficiently, prevents overfitting, and performs well on datasets of different sizes.

For a given dataset with *n* examples and *m* features $D \, = \, \{ f(x_{i} , \, y_{i} )\} \, (\left| D \right| = \, n, \, x_{i} \in R^{m} , \, y_{i} \in R)$, XGB consists of an ensemble of *K* classification and regression trees (CARTs). The final prediction is formulated as follows^31^:

5 $$\hat{y}_{i} = \sum\limits_{k = 1}^{K} {f_{k} (x_{i} ),} \, f_{k} \in F$$

$\hat{y}_{i}$ is the final predictive value, *F* is the list of CARTs, and $f_{k} (x_{i} )$ is the function of input in the *k*-th decision tree. In the XGB, the objective function consists of two components: regularization and training error, which are defined as follows^31^:

6 $$X_{obj} = \sum\limits_{i = 1}^{n} {l(y_{i} ,\hat{y}_{i} )} { + }\sum\limits_{k = 1}^{K} {\Omega (f_{k} )}$$

where $\sum\limits_{i = 1}^{n} {l(y_{i} ,\hat{y}_{i} )}$ calculates the difference between the predicted value and the observed value of the loss function. $\sum\limits_{k = 1}^{K} {\Omega (f_{k} )}$ calculates the regularization component, which is:

7 $$\Omega (f_{k} ) = \gamma T + \frac{1}{2}\lambda \left\| w \right\|^{2}$$

where $\gamma$ is the leaf penalty coefficient, *T* is the total number of a leaf node, $\lambda$ guarantees that the scores of a leaf node are not too large, and *w* is the scores of a leaf node. XGB employs the gradient boosting strategy, appends one new tree at each iteration, and modifies the preceding test results by fitting the residuals of the previous prediction:

8 $$y_{i}^{(K)} = \sum\limits_{i = 1}^{K} {f_{k} (x_{i} )} = \hat{y}_{i}^{K - 1} + f_{K} (x_{i} )$$

Integrating Eq. (1) and (2), the objective function for the *t*-th tree can be written as^31^:

9 $$L^{(K)} = \sum\limits_{i = 1}^{n} {l(y_{i} ,\hat{y}_{i}^{(K - 1)} + f_{K} (X_{i} ))} + \Omega (f_{k} )$$

Taking the Taylor expansion of the loss function up to the second order, Eq. (9) can be approximated as follows:

10 $$L^{(K)} = \sum\limits_{i = 1}^{n} {[l(y_{i} ,\hat{y}_{i}^{(K - 1)} + f_{K} (X_{i} )) + \frac{1}{2}h_{i} f_{K}^{2} (X_{i} )] + \Omega (f_{k} )}$$

11 $$\begin{aligned} X_{{obj}} = & \sum\limits_{{i = 1}}^{n} {\left[ {g_{i} f_{K} (x_{i} ) + \frac{1}{2}h_{i} f_{K}^{2} (x_{i} )} \right] + } \Omega (f_{k} ) \\ = & \sum\limits_{{i = 1}}^{n} {\left[ {g_{i} w_{q} (x_{i} ) + \frac{1}{2}h_{i} w_{q}^{2} (x_{i} )} \right] + } \Omega (f_{k} ) + \lambda T + \frac{1}{2}\lambda \sum\limits_{{j = 1}}^{T} {w_{j}^{2} } \\ = & \sum\limits_{{j = 1}}^{n} {\left[ {\left( {\sum\nolimits_{{i \in I_{j} }} {g_{i} } } \right)w_{j} + \frac{1}{2}\left( {\sum\nolimits_{{i \in I_{j} }} {h_{i} } + \lambda } \right)w_{j}^{2} } \right] + } \lambda T \\ \end{aligned}$$

where $g_{i} = \partial \hat{y}^{K - 1} l(y_{i} ,\hat{y}^{K - 1} )$ and $h_{i} = \partial^{2} \hat{y}^{K - 1} l(y_{i} ,\hat{y}^{K - 1} )$ are the first and second-order gradient statistics of the loss function.

The optimal weight $w_{j}$ of leaf j, and the objective function of a tree can be written as follows:

12 $$w_{i} = - \frac{{G_{i} }}{{H_{i} + \lambda }}$$

where $$G_{i} = \sum\nolimits_{{i \in I_{j} }} {g_{i} }$$ and $$H_{i} = \sum\nolimits_{{i \in I_{j} }} {h_{i} } + \lambda$$.

13 $$w_{j} = - \frac{1}{2}\sum\limits_{j = 1}^{T} {\frac{{G_{j} }}{{H_{j} + \lambda }}} + \gamma T$$

the weak fitting model will be intensified as follows:

14 $$f_{k} (x_{i} ) = f_{k - 1} (x_{i} ) + \sum\limits_{j = 1}^{T} {w_{j} .\eta }$$

where $\eta$ is the learning rate. XGB appends new trees at each iteration by continuously dividing features. Appending a new tree to the model is learning a new function $f_{k} (X,\theta_{k} )$ to fit the residual of previous prediction. Once K trees are learned, the strong fitting model $F(x_{i} )$ used to predict:

15 $$F(x_{i} ) = f_{0} (x_{i} ) + \sum\limits_{k = 1}^{K} {\sum\limits_{j = 1}^{T} {w_{j} .\eta } }$$

where, *F(x*_*i*_*)* is the strong-fitting model.

**Figure 2:**
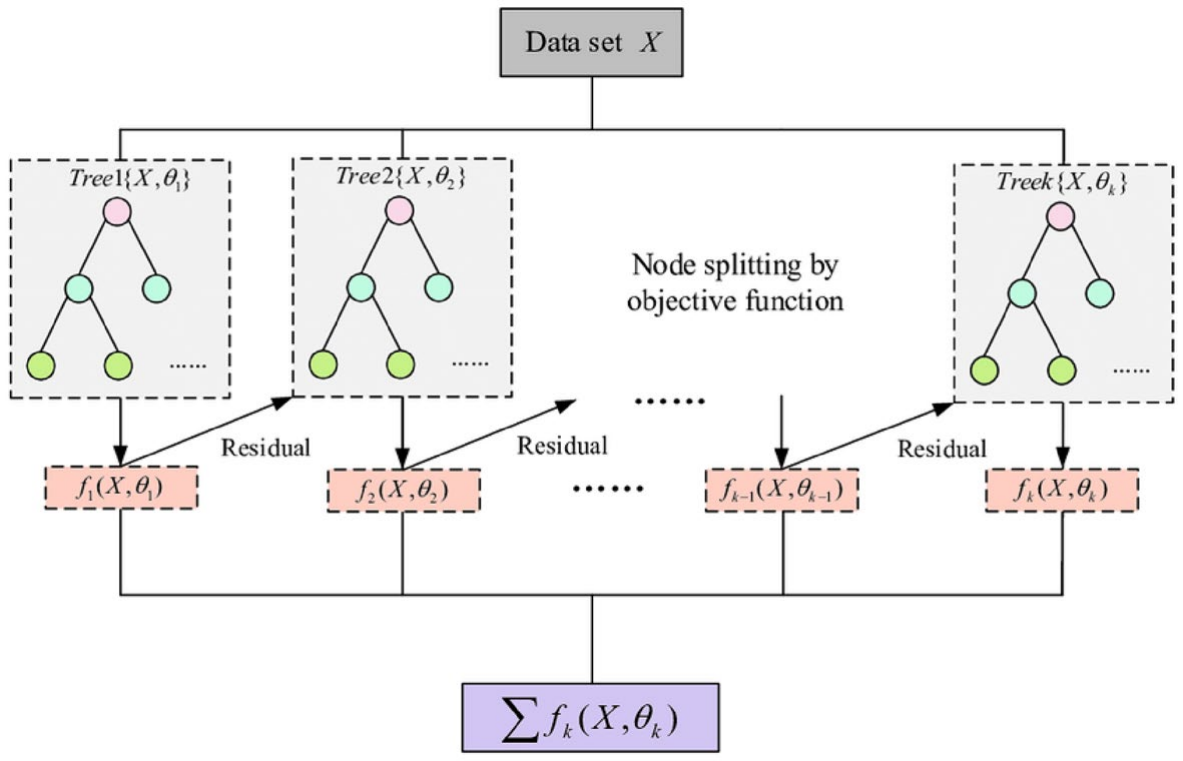
A big picture of the XGB method.

Figure 2 shows the working principle of XGB.

Since the hyper-parameters of XGB are often set empirically, optimal tuning of parameters is essential for designing robust XGB. In this paper, we used the LSHADE algorithm to tune the XGB parameters including the number of decision trees (*K*), learning rate ($\eta$), maximum depth (*md*), minimum child weight (*mcw*), gamma value ($\gamma$), sub-sample (*ss*). Table 2 lists the XGB parameters and their range used in the implementation.

**Table 2:** The parameters of the XGB algorithm that need to be tuned.

Parameter	Range
Number of decision trees (*K*)	$[1,\infty ]$
Learning rate ($\eta$)	[0, 1]
Gamma ($\gamma$)	$[0,\infty ]$
Sub sample (*ss*)	$[1,\infty ]$
Maximum depth (*md*)	$[0,\infty ]$
Minimum child weight (*mcw*)	$[0,\infty ]$

### LSHADE

Success-history-based parameter adaptation for differential evolution (SHADE)^34^ is an adaptive evolutionary optimization strategy. LSHADE^35^ enhances SHADE with a linear population size reduction technique, which gradually reduces the size of the population using a linear function. LSHADE starts its optimization process with a randomly generated population of real parameter vectors. The algorithm repeats a process of trail vector generation and selection until some termination conditions are satisfied.

### LSHADE-XGB (LXGB)

**Figure 3:**
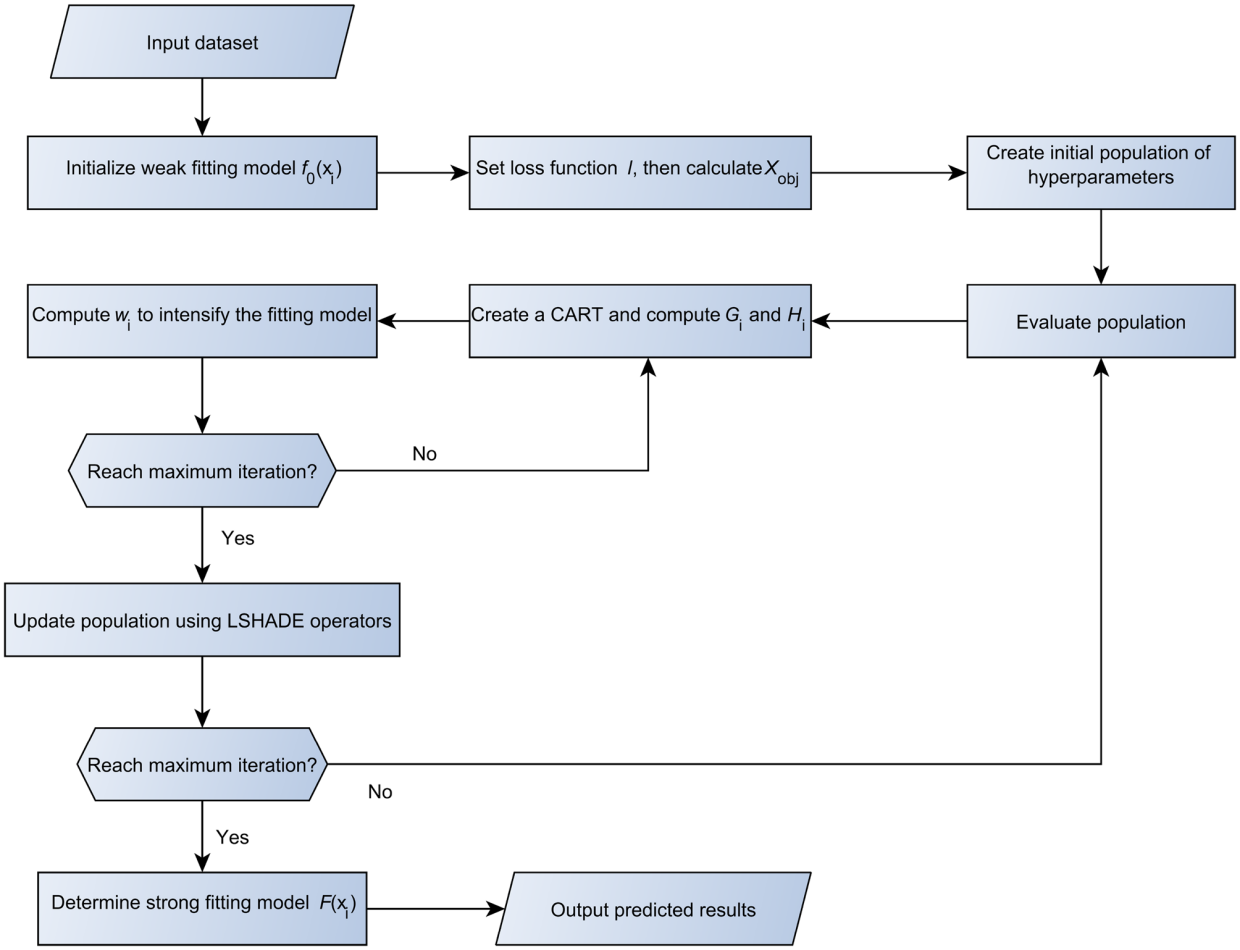
Principle of the LXGB algorithm.

The incentive mechanism of LXGB is to improve the classification performance of XGB by integrating the LSHADE optimization algorithm with XGB. Figure 3 shows the working principle of the LXGB algorithm.

### Assessment metrics

RMSE, RRMSE and NSE metrics were used to evaluate the performance of LXGB approach (Eqs. 16–18).

16 $$RMSE = \sqrt {\frac{1}{n}\sum\limits_{i = 1}^{n} {\mathop {(\mathop Y\nolimits_{i} - \mathop X\nolimits_{i} )}\nolimits^{2} } }$$

17 $$NSE = 1 - \frac{{\sum\limits_{i = 1}^{n} {\mathop {(\mathop Y\nolimits_{i} - \mathop X\nolimits_{i} )}\nolimits^{2} } }}{{\sum\limits_{i = 1}^{n} {\mathop {(\mathop Y\nolimits_{i} - \overline{X} )}\nolimits^{2} } }}$$

18 $$RRMSE = \frac{RMSE}{{\sum\limits_{i = 1}^{n} {\mathop Y\nolimits_{i} } }}$$

RMSE: Root mean square error; NSE: Nash–Sutcliffe model efficiency coefficient; RRMSE: Relative root mean square error.

Where *X*_*i*_ is the predicted values, *Y*_*i*_ is the observed values, and $\overline{X}$ is the average of *X*.

## Results and discussion

The *C*_*d*_ of PCLW1 and PCLW2 weirs was estimated using the hybrid LXGB approach. At first, all available data were normalized to remove or correct outliers^36^.

19 $$X_{n} = \frac{{X - X_{\min } }}{{X_{\max } - X_{\min } }}$$

where *X*_*min*_ is the minimum data, *X* represents the raw data, *X*_*max*_ is the maximum data, and *X*_*n*_ is the normalized data.

The ratio of the weir length to the weir height (*L/W*), the ratio of the channel width to the weir height (*B/W*), the ratio of the weir thickness to weir height (*t/W*), the number of cycles (*N*), the radius to the weir height (*R/W*), the ratio of the straight section between the weir curves length to the weir height (*S/W*), the ratio of the, the ratio of the hydraulic head to the weir height (*H/W*), were considered as input parameters of the LXGB approach. 132 datasets, including geometric and hydraulic parameters, were selected. The data were randomly divided into two parts: 80% (106 data) for training the model and 20% (26 data) for testing it.

**Figure 4:**
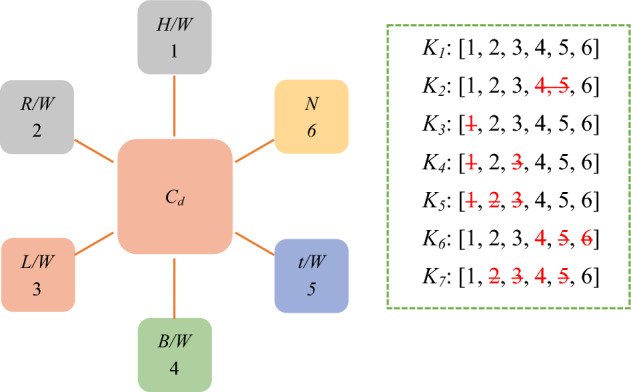
Combination of input variables (PCLW1 model).

**Figure 5:**
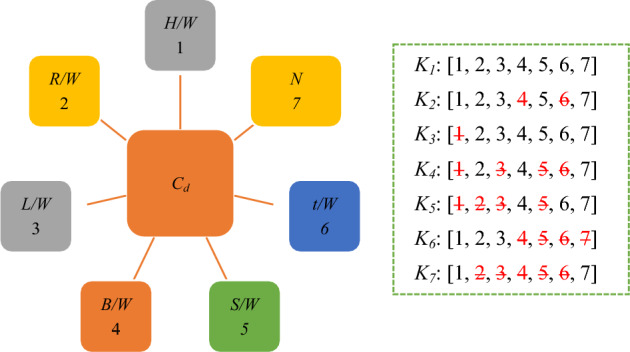
Combination of input variables (PCLW2 model).

Seven models with different variables were examined to introduce the most influential input parameters in estimating the *C*_*d*_ of PCLW1 and PCLW2 weirs. Tables 3 and 4 and Figs. 4 and 5 present various input variables.

**Table 3:** Combinations of the variables of weir in the PCLW1 plan.

Model	Input variables
*K*_1_	*H/W*, *L/W*, *R/W*, *B/W*, *t/w*, *N*
*K*_2_	*H/W*, *L/W*, *R/W*, *N*
*K*_3_	*L/W*, *R/W*, *B/W*, *t/W*, *N*
*K*_4_	*R/W*, *B/W*, *t/W*, *N*
*K*_5_	*B/W*, *t/W*, *N*
*K*_6_	*H/W*, *R/W*, *L/W*
*K*_7_	*H/W*, *N*

**Table 4:** Combinations of the variables of weir in the PCLW2 plan.

Model	Input variables
*K*_1_	*H/W*, *L/W*, *R/W*, *B/W*, *S/W*, *t/w*, *N*
*K*_2_	*H/W*, *L/W*, *R/W*, *S/W*, *N*
*K*_3_	*L/W*, *R/W*, *B/W*, *t/W*, *S/W*, *N*
*K*_4_	*R/W*, *B/W*, *t/W*, *N*
*K*_5_	*B/W*, *t/W*, *N*
*K*_6_	*H/W*, *R/W*, *L/W*
*K*_7_	*H/W*, *N*

**Figure 6:**
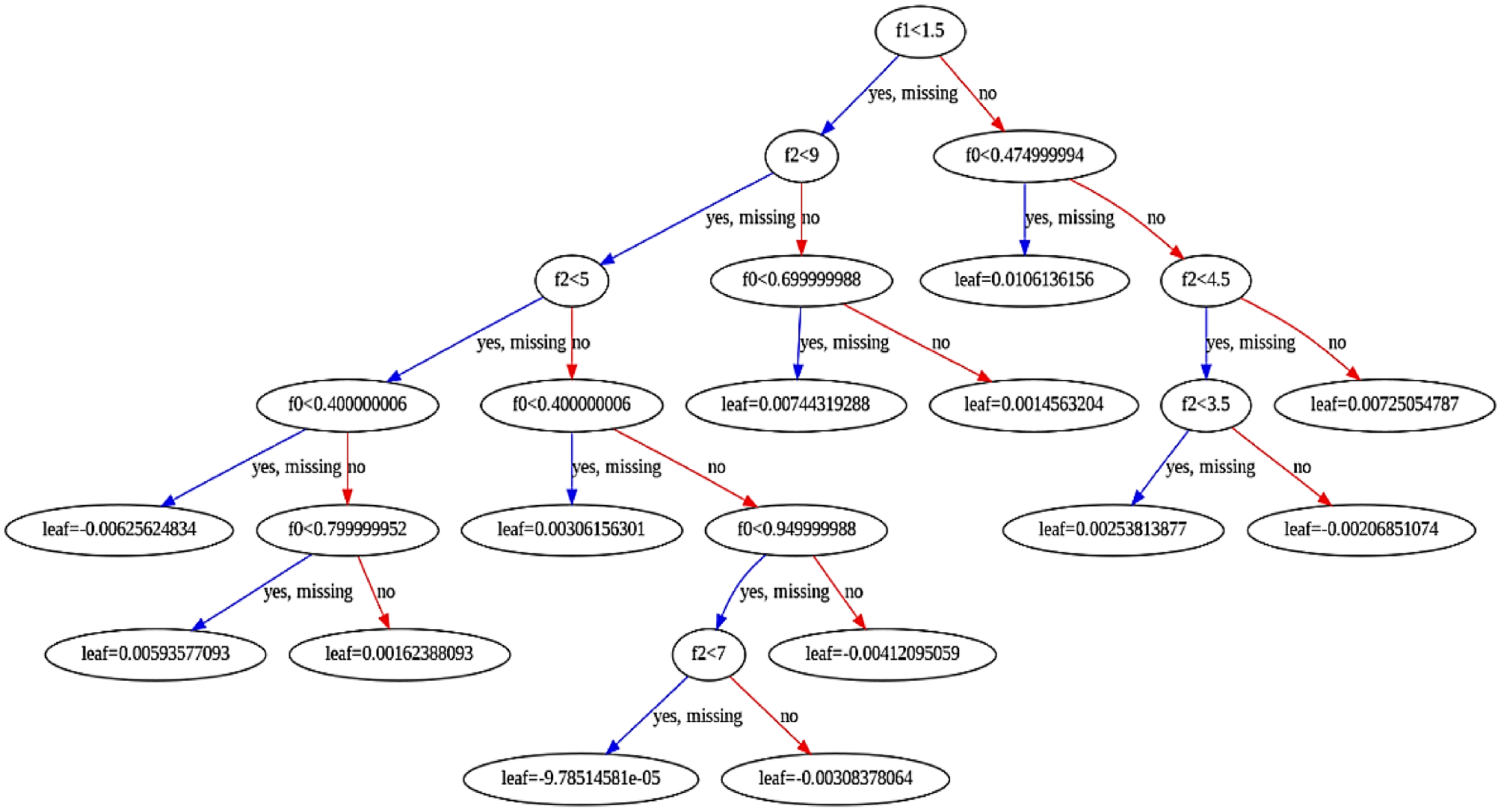
Structure of the tree generated by LXGB algorithm.

In Tables 5 and 6, the evaluation criteria for different input variables to estimate the *C*_*d*_ are presented. A part of the modeling process by the LXGB approach is presented in Fig. 6.

**Table 5:** Performance of LXGB on the PCLW1 plan with different combination models.

Model	Train			Test		
	RMSE	RRMSE	NSE	RMSE	RRMSE	NSE
*K*_1_	0.025	0.026	0.963	0.031	0.032	0.939
*K*_2_	0.013	0.015	0.973	0.017	0.019	0.952
*K*_3_	0.044	0.044	0.948	0.047	0.048	0.925
*K*_4_	0.082	0.082	0.830	0.085	0.086	0.815
*K*_5_	0.092	0.094	0.801	0.010	0.106	0.770
*K*_6_	0.008	0.009	0.991	0.013	0.015	0.972
*K*_7_	0.036	0.037	0.952	0.044	0.045	0.928

**Table 6:** Performance of LXGB on the PCLW2 plan with different combination models.

Model	Train			Test		
	RMSE	RRMSE	NSE	RMSE	RRMSE	NSE
*K*_1_	0.028	0.029	0.963	0.033	0.034	0.935
*K*_2_	0.016	0.018	0.973	0.019	0.020	0.948
*K*_3_	0.047	0.049	0.948	0.049	0.051	0.922
*K*_4_	0.083	0.085	0.830	0.087	0.089	0.810
*K*_5_	0.093	0.095	0.801	0.010	0.112	0.766
*K*_6_	0.009	0.012	0.991	0.015	0.012	0.970
*K*_7_	0.038	0.040	0.952	0.047	0.047	0.925

The results show the accuracy of the presented LXGB approach in estimating the *C*_*d*_ of PCLW1 and PCLW2 models of PCLW. Mahmoud et al.^37^ concluded that the ANFIS-PSO and MLP-FA (multi-layer perceptron and firefly optimization algorithm) methods are the most accurate in estimating the *C*_*d*_ of triangular labyrinth weirs, respectively. In a similar study, Majediasl and Fuladipanah^38^ concluded that the SVM model produces the most exact results in predicting the *C*_*d*_ of labyrinth weir with *RMSE* = 0.0118. Shafiei et al.^21^ reported that the ANFIS-FFA model is quite accurate in estimating the *C*_*d*_ of the labyrinth weir. Karami et al.^10^ showed that the ELM method with *RMSE* = 0.006 has acceptable efficiency in estimating the *C*_*d*_ of the labyrinth weir. In a similar study, the effectiveness of the least-squares support vector machine-bat algorithm (LSSVM-BA) method was used to investigate the discharge of a curved labyrinth weir^39^. The results of the studies showed that the SVM-based model gave accurate results in estimating the *C*_*d*_ of the arched labyrinth weir with values of RMSE = 0.013 and R^2^ = 0.970^40^. Multi-layer perceptron neural network (MLPNN) managed to estimate the discharge over the triangular arced labyrinth weirs of RMSE = 0.00385 and R^2^ = 0.999^41^.

**Figure 7:**
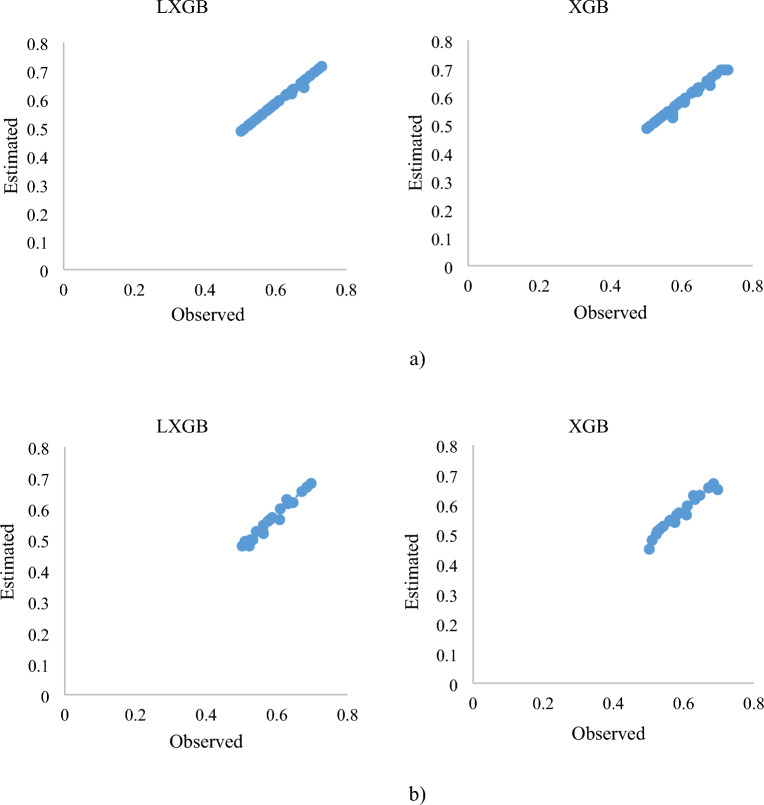
*C*_*d*_ changes of the PCLW1 (**a**) training phase, (**b**) test phase (model *K*_*6*_).

**Figure 8:**
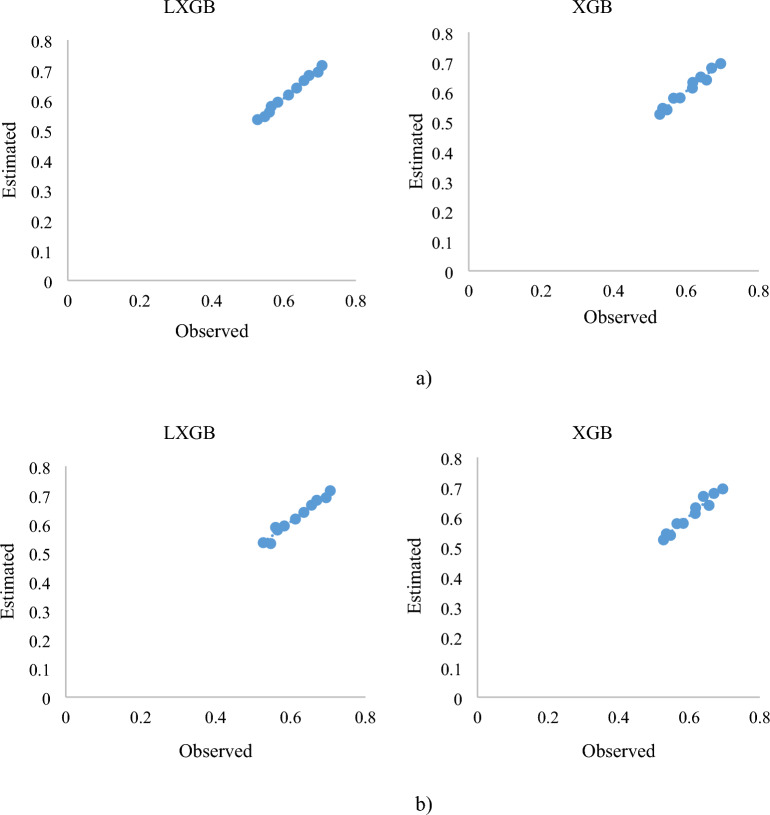
*C*_*d*_ changes of the PCLW2 (**a**) training phase, (**b**) test phase (model *K*_*6*_).

**Figure 9:**
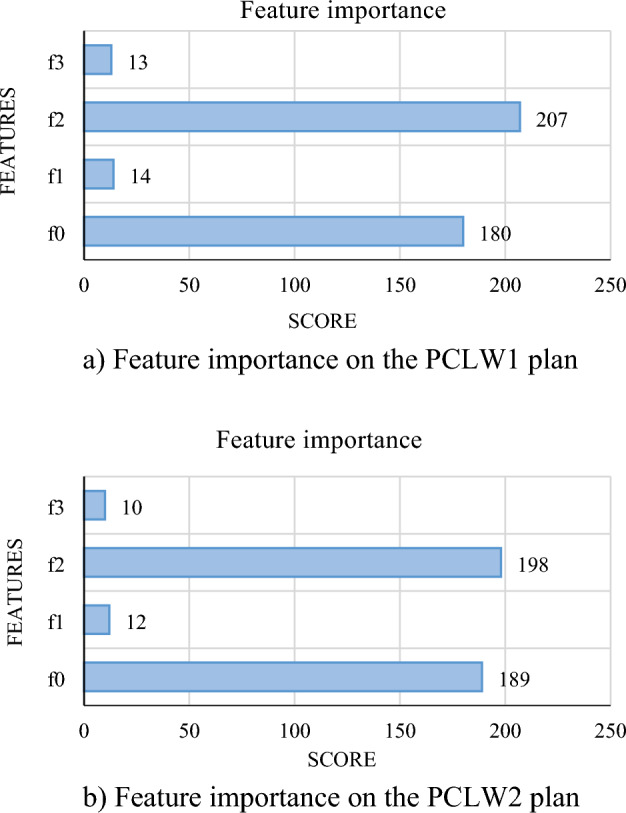
Importance of features on the *C*_*d*_estimation on the plan (**a**) PCLW1 and (**b**) PCLW2; *f*_*0*_: H/W, *f*_*1*_: R/W, *f*_*2*_: L/W, *f*_*3*_: N.

The results of the estimated and observed *C*_*d*_ of the PCLW1 and PCLW2 models of pseudo-cosine labyrinth weirs were compared in Figs. 7 and 8. According to the results, the *K*_*6*_ model with the input variables of (*R/W*), (*L/W*), and (*H/W*), had the optimal values of statistical indicators. The *C*_*d*_ of PCLW1 and PCLW2 weirs increases with the increase of the weir height. In a similar study, it was concluded that with the increase in the weir height, the *C*_*d*_ of the triangular duckbill labyrinth weir increases, which is in agreement with the results of the present study^7^. The increase in the effective length of the labyrinths at a specified width, due to the radius increases of PCLW1 and PCLW2 weirs causes an increase in the Cd. The studies showed that increasing the radius causes a reduction in eddy flows, turbulence, and a sudden increase in water height during the weir^39, 40, 42^. The results of the investigations showed that with the increase of R/W, the *C*_*d*_ increases in the arched labyrinth weir, which is consistent with the results of the present study^41^. Also, the *K*_*2*_ model (*H/W, L/W, R/W, N*) is in the second rank, which shows that length, weir height, radius, and the number of cycles have a more significant impact on *C*_*d*_ of PCLW1 and PCLW2 weirs. By increasing the number of labyrinth weir cycles, discharge and Cd increase, which is consistent with the results of the present study^40, 43^. Figure 9 shows the importance of the influential input parameters in estimating the *C*_*d*_ of PCLW.

Emami et al.^44^ predicted the *C*_*d*_ of a curved plan labyrinth weirs using the WOA-ANFIS method, and the input parameters *H/W* and *θ* (weir arc angle) were introduced as the most effective parameters in estimating the *C*_*d*_. Majediasal and Fuladipanah^38^, investigated the support vector machine (SVM) method for *C*_*d*_ of sharp-crested triangular labyrinth weirs and concluded that the input combination, including geometric parameters (*θ, h/w, L/B*), has the best results. Mohammadi et al.^45^ reported that the parameters *H*_*t*_*/P*, *W/P* (the ratio of the weir width to the height), *R/W*,* W/LC* (the ratio of the weir width to the effective length) as input variables have the most accuracy and efficiency in estimating the *C*_*d*_ of U-shaped labyrinth weirs. Haghiabi et al.^46^ indicated the *C*_*d*_ of triangular labyrinth weirs using the ANFIS system and concluded that the ANFIS has a proper implementation in *C*_*d*_ estimation. Studies showed that the *H/W* parameter is the most influential parameter on the *C*_*d*_ of a labyrinth and arced labyrinth weirs^47^.

Table 7 compares the performances of the XGB and LXGB on the test dataset. The results show the superiority of the LXGB compared with the XGB algorithm in terms of performance measures. This issue proves that combining the LSHADE with XGB improves the estimation performance.

**Table 7:** Performance evaluation of the XGB and LXGB algorithms on test dataset.

Method	Plan	R^2^	RMSE	NSE
XGB	PCLW1	0.953	0.025	0.940
LXGB	PCLW2	0.970	0.013	0.972
XGB	PCLW1	0.954	0.027	0.941
LXGB	PCLW2	0.970	0.015	0.970

In Table 8, the values of the evaluation criteria for estimating the *C*_*d*_ of labyrinth weirs with different plans have been compared with the results of other studies. The results for LXGB are generated with the PCLW1 plan. The comparisons show the appropriate accuracy of the LXGB approach in estimating the *C*_*d*_ of labyrinth weirs with *R*^*2*^ = 0.97 and *RMSE* = 0.014.

**Table 8:** Performance evaluation of the LXGB approach and similar methods.

Method	R^2^	RMSE	NSE
SAELM	0.950	–	0.960
ANFIS-FFA	0.940	0.039	0.901
GEP	0.967	0.017	0.780
ANN	0.920	0.045	–
LXGB	0.970	0.014	0.971

## Conclusion

This study introduces a novel design for labyrinth weirs called pseudo-cosine labyrinth weirs (PCLW). The LXGB was used to estimate the *C*_*d*_ of the PCLW weir. Seven models with different combinations of appropriate input parameters were introduced. A proper model was defined by analyzing the estimation results. The superior model estimates *C*_*d*_ by considering input parameters *H/W*,* R/W*, and *L/W*. LXGB was achieved in estimating the *C*_*d*_ of PCLW overflows by obtaining values of *R*^*2*^ = 0.971, *RMSE* = 0.014, and *NSE* = 0.97. The results demonstrated that the proposed LXGB algorithm generated more significant results than previous studies in estimating the *C*_*d*_ of labyrinth weirs. Such a cost-effective prediction model may have significant practical application, as it can be an economical alternative to the expensive laboratory solution, which is costly and time-consuming. The proposed model is useful to correct the design of water transfer systems.
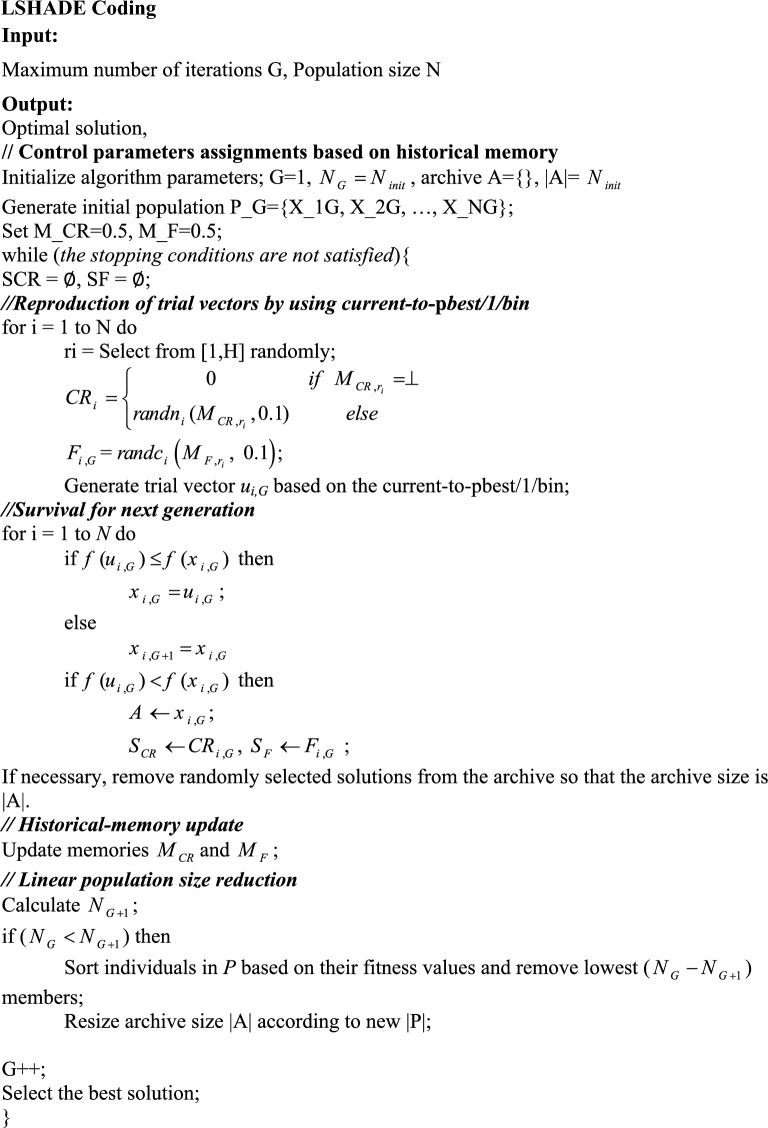


## Data Availability

The datasets generated and/or analyzed during the current study are not publicly available but are available from the corresponding author on reasonable request.
